# American Medical Literature

**Published:** 1850-01

**Authors:** 


*American Medical Literature.*—The North Western Medical Journal states that the article by Dr. Brainard, on the treatment of spina bifida by iodine injections, which our readeis will find in the eclectic department of a former number of this Journal, attracted but little notice in this country prior to its having been copied into a Parisian Medical Journal, and afterward re-translated by D. W. Yandell, of Louisville. In the latter form it is going the rounds of the American journal.

